# KMT2A重排急性淋巴细胞白血病复发后谱系转换1例报告并文献复习

**DOI:** 10.3760/cma.j.cn121090-20260228-00114

**Published:** 2026-08

**Authors:** 卓青 乔, 艳萍 石, 亮钉 胡

**Affiliations:** 解放军总医院血液病医学部，北京 100071 Senior Department of Hematology, Chinese PLA General Hospital, Beijing 100071, China

## Abstract

为提高对谱系转换（lineage switch, LS）这一现象的认识，回顾性分析1例KMT2A重排急性淋巴细胞白血病（ALL）复发后出现LS的病例资料，并对相关文献进行复习。患者，女，23岁，诊断ALL（KMT2A::AFF1阳性），巩固化疗期间复发，予以含有贝林妥欧单抗方案的化疗再次缓解后，桥接异基因造血干细胞移植，移植后2个月余广泛髓外复发，髓外肿物病理免疫组化显示，CD19表达部分缺失，并出现MPO表达，但仍保留KMT2A重排；行靶向CD19的嵌合抗原受体T细胞治疗后，再次髓外复发时，CD19表达完全缺失，MPO阳性，诊断为髓系肉瘤。最终患者放弃治疗，返回当地。抗原靶向免疫疗法后，LS作为一种耐药和适应性机制，需引起临床重视。

## Abstract (en)

To enhance the understanding of lineage switch（LS）, we retrospectively analyzed the clinical data of a patient with KMT2A-rearranged acute lymphoblastic leukemia（ALL）who underwent a LS at relapse and reviewed the relevant literature. The patient, a 23-year-old woman, was initially diagnosed with ALL（KMT2A::AFF1-positive）. She experienced a relapse during consolidation chemotherapy and achieved a second remission following chemotherapy containing blinatumomab, subsequently bridging to allogeneic stem cell transplantation. Approximately 2 months after transplantation, extensive extramedullary relapse occurred. Immunohistochemical analysis of the extramedullary mass revealed a partial loss of CD19 expression and emergence of MPO expression, whereas the KMT2A rearrangement was retained. After receiving CD19-CAR-T therapy, the patient experienced another extramedullary relapse, at which time complete loss of CD19 expression and positivity for MPO were observed, leading to a diagnosis of myeloid sarcoma. Ultimately, the family opted to discontinue treatment and the patient was discharged to receive care locally. Following antigen-targeted immunotherapy, LS, as a potential mechanism of drug resistance and adaptation, warrants increased clinical attention.

KMT2A重排（KMT2Ar）作为早期白血病发生的独特驱动因素，可导致原始细胞同时具有髓系和淋系的免疫表型特征，故曾被称为混合谱系白血病（mixed lineage leukemia, MLL）基因，可出现在急性髓系白血病（AML）、急性淋巴细胞白血病（ALL）或混合表型急性白血病（MPAL）中。伴KMT2Ar的ALL化疗耐药、复发率高，预后差，5年总生存率不足20％[1]。靶向免疫治疗及细胞治疗，彻底改变了复发/难治性ALL的治疗格局。但这种高强度淋系靶向治疗后，部分患者发生淋系向髓系白血病的谱系转换（lineage switch, LS），作为一种耐药和适应性机制[2]，成为新的治疗挑战。现报道1例KMT2Ar ALL靶向免疫治疗后出现LS的诊疗经过并进行文献复习，提高对此现象的认识。

## 病例资料

患者，女，23岁，行骨髓穿刺确诊为KMT2A::AFF1融合基因阳性ALL。初诊时，骨髓流式细胞术免疫分型显示：74％的异常细胞群表达HLA-DR、CD15、CD19、CD22、CD38、CD58、CD123、cCD79a及TdT。经长春地辛、伊达比星、环磷酰胺、培门冬酶、泼尼松组成的VICLP方案诱导化疗后达到完全缓解（CR），随后接受巩固治疗。于第2个疗程巩固治疗后出现复发。复发后，予以长春地辛、盐酸米托蒽醌、环磷酰胺、泼尼松组成的VMCP联合贝林妥欧单抗方案进行再诱导化疗，再次获得CR。此后桥接其父为供者的半相合外周血造血干细胞移植。+17 d粒系植入，+30 d血小板植入。移植后1个月复查骨髓，提示完全分子生物学缓解及完全供者型植入。移植后2个月余，患者出现广泛髓外复发，临床表现为腮腺肿物、多发皮肤硬性结节、左侧眶周肿物。皮肤结节活检病理示：真皮层及皮下脂肪组织见形态相对一致的异型淋巴样细胞浸润，细胞中等大小，胞质稀少，部分可见单个核仁。免疫组化示：CD3（−）、CD20（−）、CD19（少部分+）、PAX-5（−）、CD79a（−）、CD34（−）、CD99（−）、CD10（−）、TDT（−）、CD117（−）、CD38（部分+）、CD138（−）、MPO（+）、CD33（−）、Lysozyme（少部分+）、CD4（+），CD56（−）、CD123（−）、Ki-67（约90％+）、P53（90％+）、Bcl-2（弥漫+）、Bcl-6（−）、c-myc（80％+）、CD7（−）；原位杂交结果：EBER（−）。荧光原位杂交（FISH）检测显示皮肤结节中XY信号表达率为1％，KMT2Ar阳性率为98％，证实该异型淋巴细胞来源于患者且携带与初诊白血病一致的KMT2Ar；而非来源于供者、在EB病毒刺激下引起的移植后淋巴增殖性疾病。腮腺肿物穿刺与皮肤结节活检病理形态、免疫组化及FISH结果一致。后续予以靶向CD19的嵌合抗原受体T细胞（CAR-T细胞）治疗，上述髓外肿物曾一过性消退。CAR-T细胞治疗后1个月再次出现髓外复发。再次皮肤活检病理示：纤维组织间见异常细胞增生，细胞中等大小，胞质少，核呈椭圆或略不规则，染色质细腻，部分可见小核仁。免疫组化：Bcl-2（90％+）、CD3（−）、CD7（−）、CD10（−）、CD13（−）、CD19（−）、CD20（−）、CD22（−）、CD33（<5％+）、CD34（−）、CD38（90％+）、CD79a（−）、CD99（+）、CD117（−）、CD138（−）、C-myc（90％+）、Ki-67（90％+）、MPO（部分弱+）、PAX5（−）、TdT（−），符合髓系肉瘤诊断。此后尝试CD38单克隆抗体治疗2次，疗效不佳。拟申请menin抑制剂临床试验但须等待入组。最终患者放弃积极治疗，返回当地。

## 讨论及文献复习

1. LS的定义：目前关于LS的较大规模研究主要来自国际协作项目EVOLVE[3]。该项目汇集了来自8个国家、43个机构或团体的75例LS病例，并依据严格标准对“LS”进行定义，即在保留原始细胞遗传学或分子标志的同时，出现从一种谱系向另一种谱系的形态学及免疫表型转换，最常见为B-ALL向AML的转化。

2. KMT2Ar急性白血病患者是发生LS的高危人群：以往基于FAB谱系分型标准，白血病复发时LS的发生率初步估计约为6.7％。然而，随着1984年以来免疫表型检测技术的发展及当前WHO采用的更严格的谱系判定标准，这一数据可能被高估。多数LS是从ALL转为AML，但也有AML转为ALL的个案报道[4]–[5]。此类现象最常见于携带KMT2Ar的年轻患者。Lamble等[6]报道，在接受CAR-T细胞治疗的163例B-ALL患者中，有12例发生了LS，其中75％携带KMT2Ar。除KMT2Ar外，研究亦发现其他细胞遗传学和分子异常与免疫治疗后LS相关，如BCR::ABL1[7]、TCF3::ZNF384融合基因或ZNF384重排[8]、DUX4重排[9]、PAX5 P80R突变[9]、CRLF2重排[10]、TP53突变[11]等。这些亚型在初诊、治疗期间或复发时易表达髓系标志物，因此有必要加强对这些患者群体的LS监测。

3. LS时髓外复发的特点：Aldoss等[2]和He等[12]报道2例KMT2A::AFF1易位的ALL患者复发后，接受靶向治疗后，分别发生胸部皮下髓系肉瘤和乳腺的髓系肉瘤。EVOLVE项目[3]报道的LS最常在骨髓中被发现（65.7％），亦可见孤立性中枢神经系统浸润（4.3％）、非中枢神经系统髓外病变（5.7％）或骨髓合并髓外病变受累（22.9％），即高达32.9％的患者LS时出现髓外病变。髓外逃逸和CD19抗原丢失是ALL患者接受贝林妥欧单抗治疗后复发的特征模式[2]。

4. 发生LS的机制：LS的确切机制尚未完全阐明，目前认为可能涉及多种生物学通路。现有解释主要基于以下几种假说：

第一，“谱系可塑性”假说。髓系与淋系细胞均起源于共同的双潜能B/巨噬祖细胞。LS后的白血病细胞与原始克隆之间存在明确的克隆相关性。通过比较诊断时与治疗后样本的免疫球蛋白重链基因重排、细胞遗传学或分子特征，可证实两者的同源性。复发时细胞可能获得新的遗传学异常，但仍保留原有的TCR/IGH重排或分子标志。Coorens等[13]报道了1例3岁B-ALL女性患儿，在化疗期间，经历2次与初诊白血病表型一致的复发后，经抗CD19 CAR-T细胞及贝林妥欧单抗治疗获得短期缓解。然而，7个月后进展为AML，且对后续治疗耐药。研究者通过对6个时间点的8份骨髓或外周血样本进行全基因组测序，分析各类变异发现，AML克隆源于初诊ALL，但并非由最近一次复发的淋系克隆直接转化，而是从白血病遗传多样性背景中预先存在的谱系演化而来。该结果提示，具备LS潜能的癌细胞可能以隐匿状态持续存在，在耐受治疗的同时不断累积突变，反映出白血病克隆群体内存在高度的遗传异质性与适应性进化潜能。

第二，治疗压力下表观遗传学改变及微环境调节因子介导的细胞重编程。Wölfl等[14]报道1例ALL患儿在异基因造血干细胞移植后早期复发，接受贝林妥欧单抗治疗仅11 d后转化为AML；而停药观察7 d后，白血病表型竟自发逆转为原来的CD19⁺淋巴细胞表型。该案例揭示了早期祖细胞在免疫压力下具有高度的可塑性。CD19靶向治疗初期常伴随强烈的细胞因子释放。研究显示炎症因子（特别是IL-6）可能促使淋系克隆向髓系分化[12],[14]–[17]。Janssens等[18]研究揭示KMT2A致癌融合蛋白（癌蛋白）通过表观遗传学机制，使KMT2Ar白血病能够逃逸靶向治疗。他们通过对1例接受淋系靶向治疗后发生淋系到髓系LS的样本进行分析发现，在癌蛋白表达水平降低时，其靶基因网络发生改变，癌蛋白会转向非常规调控程序并促使细胞倾向髓系分化。单纯CD19丢失并不足以导致LS，这进一步支持KMT2Ar背景下存在更复杂的表观遗传细胞重编程机制。

第三，治疗诱导的克隆选择。多项研究表明，部分白血病在初诊时即存在双谱系或混合表型的原始细胞群[4]。KMT2Ar婴儿ALL中已观察到显著的克隆异质性[19]–[20]，诱导治疗结束时高MRD患者，其白血病细胞更常共表达髓系标志物，提示ALL样诱导方案可能筛选出具有“AML样”特征的亚克隆[21]。CD19靶向药物如贝林妥欧单抗或CAR-T细胞治疗，可有效清除CD19⁺淋系克隆，从而使得原本占比较低的髓系表型或双表型亚克隆获得增殖优势，最终表现为LS[14],[22]。

5. LS与混合白血病的关系：Bełdzińska-Gądek等[23]共汇总了106例发生LS的患者，多数病例（80例，75.5％）为ALL向AML转换，18例（17.0％）为AML向ALL转换，其余为MPAL转为ALL/AML，或ALL/AML转为MPAL，4例患者（3.8％）经历了2次LS（转换路径分别为：B-ALL→AML→MPAL；T-ALL→AML→T-ALL；B-ALL→AML→B-ALL；B-ALL→MPAL→AML）。其中49例患者（46.2％）携带KMT2Ar，3例携带TCF3::ZNF384重排。36例患者（34.0％）为携带KMT2Ar的B-ALL，其中2例转换为髓系肉瘤。EVOLVE项目[3]还报道了10例谱系漂移（表现为表型不完全或暂时性改变），“谱系漂移”适用于以下情况：细胞表面抗原表达发生演变，导致与先前表型相比出现显著变化，但不足以支持疾病重新分类（例如，CD19丢失但保留CD22，并获得CD33，但未出现其他髓系标志物）。我们报道的此例患者，第一次髓外复发时，皮肤病理及腮腺组织活检病理：MPO阳性，CD38阳性，CD117阴性，CD33阴性，CD19少部分阳性。从免疫组化可以看出，此患者B-ALL的表型正在丢失，AML的表型正在显现，髓系特征性的MPO阳性，但CD117及CD33阴性，CD13未做，但因受限于病理免疫组化有限的标记，无足够的证据把这一中间状态归为MPAL，只能归为急性未分化型白血病（AUL）。

6. LS后的治疗：LS后的患者预后极差，治疗方案多样且缺乏统一标准，治疗效果通常有限、短暂或不完全，这可能与持续治疗所导致的内在或获得性化疗耐药有关。EVOLVE项目[3]数据显示，在70例B-ALL转为AML或MPAL的LS患者中，确诊后的中位总生存期仅为4.8个月。值得关注的是，其中8例存活患者中有7例为KMT2Ar白血病，提示即使在这一高危亚群中挽救性治疗仍可能带来生存获益。目前LS的治疗策略较为多样，主要包括化疗联合靶向药物，如吉妥珠单抗[24]、维奈克拉[24]、达雷妥尤单抗[25]、奥加伊妥珠单抗[24]、menin抑制剂[26]、DOT1L抑制剂[27]等均有相关病例报道。CPX-351在LS患者中的作用仍需进一步研究来明确。Fms样酪氨酸激酶3受体（FLT3）在AML中常因突变激活，在KMT2Ar ALL中也可见野生型FLT3过度表达。基于此，Niswander等[28]开发了单价FLT3 CAR-T细胞及双特异性CD19×FLT3 CAR-T细胞。临床前研究表明，尽管LS后FLT3表面抗原密度下降，FLT3 CAR-T细胞在KMT2Ar ALL和AML的患者来源异基因移植模型中均展现出显著的白血病抑制活性。这些结果提示，FLT3 CAR-T细胞可能尤其适用于具有LS高风险的KMT2Ar ALL患者。多数长期存活的个案报道显示，患者在诱导缓解后接受了巩固性造血干细胞移植[24]，提示移植可能是实现治愈的关键环节。

移植策略的优化主要包括以下几种：移植前强化疗或新型治疗方法（免疫治疗、DOT1L抑制剂、menin抑制剂、HDAC抑制剂等）用于难治/复发患者的移植前治疗，争取达CR或MRD阴性，加强预处理，移植后增强移植物抗白血病（GVL）效应或新型疗法抢先治疗或维持等，最大程度提高移植疗效，减少移植后复发[29]。需注意的是，使用吉妥珠单抗或奥加伊妥珠单抗等药物后桥接移植时，应警惕肝窦阻塞综合征等风险。

LS是白血病治疗过程中的罕见事件，其机制尚未完全阐明，且目前缺乏标准治疗方案，患者预后普遍较差。尽管抗原靶向免疫疗法已显著提升了急性白血病的治疗水平，但髓外逃逸、靶向抗原丢失，甚至出现LS作为新的复发模式已经显现。所有接受抗原靶向免疫治疗的患者均面临LS风险，特定基因组亚型（如KMT2Ar等）的患者风险尤为突出。随着免疫治疗逐渐向一线推进，这类免疫治疗后出现LS的病例数量可能会进一步增加。在免疫治疗后，除常规多参数流式细胞术监测外，建议采用包括细胞遗传学与二代测序在内的多模式MRD追踪策略，以提高LS的检出与确诊率。推荐在白血病初诊、进展或发生LS等关键节点进行系统的基因测序与细胞遗传学分析，并建立纵向样本库，从而深入理解LS的生物学机制。目前，LS后的治疗仍具有挑战，靶向药物、去甲基化治疗等可能带来一定疗效，而异基因造血干细胞移植被认为是目前实现治愈的重要途径。
